# Digital Health Literacy and Infodemic: the impact on Italian medical students between 2019-2020: Valentina Moretti

**DOI:** 10.1093/eurpub/ckac130.063

**Published:** 2022-10-25

**Authors:** V Moretti, L Arnoldo, G Valdi, A Conte, M Masoni, MR Guelfi, F Anelli, L Brunelli

**Affiliations:** 1 Department of Medicine, University of Udine, Udine, Italy; 2 Quality and Risk Management Unit, Friuli Centrale Healthcare and University Trust, Udine, Italy; 3 Medical Directorate, Friuli Centrale Healthcare and University Trust, Udine, Italy; 4 Department of Medicine, University of Firenze, Firenze, Italy; 5 FNOMCEO, Italian Federation of Medical Professional Associations, Rome, Italy

## Abstract

**Background:**

The COVID-19 infodemic is putting pressure on public health systems to control the pandemic. With the internet and social media playing a key role in emergency communication, digital health literacy (DHL) can be considered a determinant of health. This study aims to assess the impact of infodemic on the skills of medical students, for whom low levels of DHL may affect the ability to identify the best available medical evidence.

**Methods:**

A cross-sectional web-based survey was conducted at the University of Florence (Italy) in Apr-May 2019 (pre-pandemic period) and in Nov-Dec 2020 (pandemic period) to investigate DHL skills. Two different cohorts of students, both in their first year of medical school, participated in the survey. The 8-item self-assessment tool (IT-eHEALS) with a 5-point Likert scale was used to examine DHL. The change in perception of ability between the two cohorts was examined using the Wilcoxon test.

**Results:**

A total of 329 students participated in the survey in 2019 (F: 58.1%; mean age 20.6±2.1) and 341 in 2020 (F:61.9%; mean age 19.8±2.0). In 2019, participants’ DHL level was moderate with a IT-eHEALS overall mean score (MS) of 28.4±5.8. Students had a good idea of how to find helpful health information (MS 3.9±0.8) and how to use the web for this purpose (MS 3.8±0.9), but they were less confident about the usefulness of the information they received (MS 2.9±1.1). In 2020, the medical students’ DHL level deteriorated as the overall MS of IT-eHEALS decreased to 23.4±7.2 (p < 0.01). The scores of the IT-eHEALS items were significantly lower and students indicated that they found it difficult to assess the information they found (MS 2.4±1.1; p < 0.01).

**Conclusions:**

DHL can contrast infodemic, but the latter in turn may have a negative impact on perceived DHL skills if personal knowledge base is not well structured. Training programmes for medical students as future health care providers should be reinforces to guide their practise.

**Key messages:**

- Assessing digital health literacy is the first step in directing public efforts towards empowering educational programmes to improve health literacy.
- Medical students, as future health professionals, should be able to use the best evidence to help their patients identify healthy beliefs and behaviours to manage in health emergencies.

